# Development and optimization of a rapid BOD detection method using microbial immobilized particles with polyvinyl alcohol (PVA) and diatomite modifiers

**DOI:** 10.1016/j.mex.2024.102595

**Published:** 2024-02-01

**Authors:** Huaihuai Huo, Jie Li, Amirah Hurzaid

**Affiliations:** a Biological Sciences Program, School of Distance Education, Universiti Sains Malaysia, Minden, Penang 11800, Malaysia; b School of Environmental and Municipal Engineering, Qingdao University of Technology, Qingdao 266033, China; c School of Biological Sciences, Universiti Sains Malaysia, Minden, Penang 11800, Malaysia

**Keywords:** Microbial sensor, Microbial immobilization particles, Immobilization, Embedded agent, Rapid BOD Detection Method Using Microbial Immobilized Particles

## Abstract

Biochemical oxygen demand (BOD) serves as an important indicator in water quality monitoring. It provides valuable information for studying biology and conducting environmental impact assessments, making it the preferred method for environmental applications. Currently, the most common approach for BOD monitoring is the BOD_5_ standard detection method. However, this method has several drawbacks, like a long 5-day culture time, extended detection duration, complex operations, and low reproducibility of results. To address these issues, our study introduces a rapid BOD detection method, that focused on optimizing microbial immobilized particles and their detection capabilities. The method demonstrated better detection accuracy, stability, and reproducibility, with results available in less than 8 min. Our customization includes:

• Prepared the particles using the cross-linking-embedding method by adding specific modifiers which are Polyvinyl Alcohol (PVA) and diatomite.
• Improved the detection results, reducing the overall detection error by over 10%.
• Confirmed our method’ effectiveness in rapidly detecting BOD solution prepared in the lab, outsourced BOD standard solution and actual waste water samples with high accuracy.

## Graphical abstract

**Figure:**
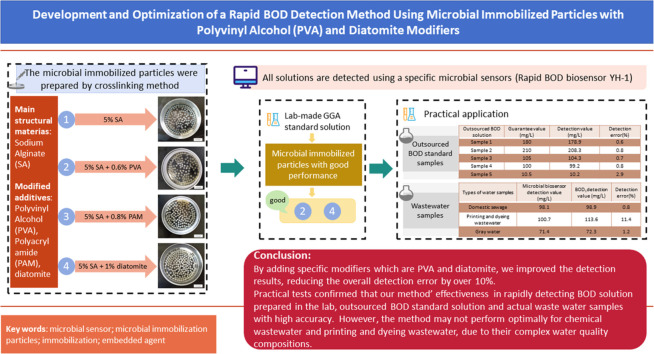

Specifications table

**Table:** 

Subject area:	Environmental Science
More specific subject area:	Environmental Monitoring
Name of your method:	Rapid BOD Detection Method Using Microbial Immobilized Particles
Name and reference of original method:	Five-Day Biochemical Oxygen Demand (BOD_5_) (APHA, 1992)
Resource availability:	We used a microbial sensing method based on BOD_5_ principles (Rapid BOD biosensor YH-1) developed by Wuxi Yuheng Environmental Technology Co., Ltd. In this experiment, we used *Bacillus subtilis* (obtained from Nantong Kaiheng Biotechnology Development Co., Ltd.). Sodium chloride (NaCl); Glucose; Glutamate; Potassium chloride (KCl); Potassium dihydrogen phosphate (KH_2_PO_4_); Disodium hydrogen phosphate (Na_2_HPO_4_·12H_2_O); Calcium chloride (CaCl_2_); Sodium alginate (SA); Polyvinyl Alcohol (PVA); Diatomite

## Method details

### Instrument for rapid BOD detection

We used a microbial sensing method based on BOD_5_ principles (Rapid BOD biosensor YH-1) developed by Wuxi Yuheng Environmental Technology Co., Ltd. Unlike traditional methods that measure the complete metabolic process, this instrument concentrates on the initial reaction and calculates the BOD value using a specific formula. It uses immobilized microbial cells for the reaction, ensuring accurate measurements and minimizing microorganism loss. This approach guarantees thorough mixing and reaction between the water sample and the tested microorganisms.

### Solutions preparation

We used *Bacillus subtilis* obtained from Nantong Kaiheng Biotechnology Development Co., Ltd. Before inoculation, *B. subtilis* subcultures were refrigerated at 4 °C, then were inoculated into a liquid medium and incubated at 25 °C with a rotation speed of 100 r/min. All solutions used were prepared using deionized water. The liquid medium was made by dissolving a 10 g of tryptone, 5 g of yeast extract, and 10 g of sodium chloride (NaCl) together. The pH was adjusted to 7.4 with sodium hydroxide (NaOH), and the volume was topped up to 1 L. For the BOD standard solution, we dissolved 136.4 mg of glucose and 136.4 mg of glutamate (previously dried at 103 °C for 2 h, and cooled to room temperature), in 100 mL to yield a BOD of 2000 ± 160 mg/L. Lower BOD concentrations were made by diluting this standard solution with deionized water. The saline (0.8%) solution was made by dissolving 8 g NaCl in deionized water and adjusting the volume to 1 L, then sterilized in a conical flask. The electrolyte solution was made by dissolving 745 mg of potassium chloride (KCl) in deionized water and adjusting the volume to 100 mL. The phosphate buffer solution (PBS) was made by dissolving 0.68 g potassium dihydrogen phosphate (KH_2_PO_4_) and 1.49 g disodium hydrogen phosphate (Na_2_HPO_4_·12H_2_O) and adjusting the volume to 1 L, yielding a phosphate buffer with a concentration of 0.005 mol/L. The cross-linking solution (6% w/w) was prepared by dissolving 6 g calcium chloride (CaCl_2_) in deionized water and adjusting the volume to 100 mL.

### Cross-linking-embedding method to immobilized microorganisms

We made spherical microbial immobilized particles by using CaCl_2_ solution to cross-link them. Then, we set up several groups with different combinations for comparative tests. Group 1 used only sodium alginate (SA) as the main material (Control group). Group 2, we added Polyvinyl Alcohol (PVA) as modified additive. Group 3 had Polyacrylamide (PAM) added to the immobilized particles, and Group 4 included diatomite. All these groups resulted in spherical-shape immobilized particles. The goal was to see whether these additives could improve the performance of the immobilized particles.

**Fig. 1:**
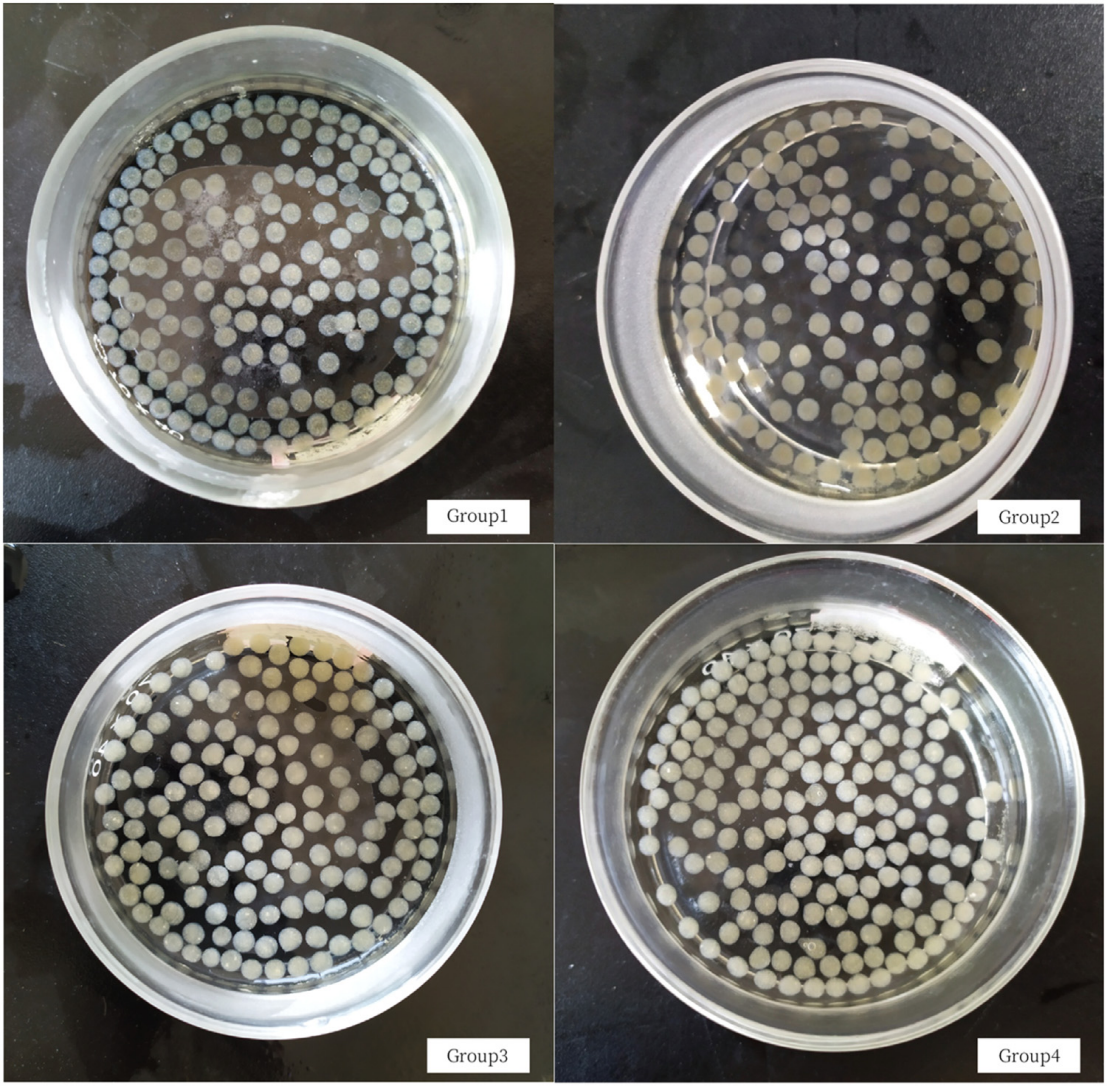
Microbial immobilized particles produced in this study. Group 1 (Control), SA; Group 2, SA + PVA; Group 3, SA + PAM; Group 4, SA + diatomite.

In the initial phase of this experiment, we screened the embedding based on particle formation characteristics, aiming for uniform size and well-defined shapes. We found that the most favorable particle formation occurred when using SA concentrations between 4% and 6% (w/w). After screening, we found that optimal particle formation happened with PVA concentrations between 0.5% and 0.7% (w/w). Similarly, PAM worked best at concentration ranging from 0.5% to 1% (w/w), while diatomite's ideal concentration was between 0.8% and 1.2% (w/w). We then selected materials combination with varying concentrations for further experiments. The particles with the highest carrying power were chosen for additional testing. The distribution of each material and corresponding photos of the embedding particles is presented in Table 1 and Fig. 1, respectively.

**Table 1:** Various materials and proportions for making the embedding agent.

Configuration	Group 1 (Control)	Group 2	Group 3	Group 4
Material	SA	SA + PVA	SA + PAM	SA + diatomite
Dosage	5%	5%+0.6%	5%+0.8%	5%+1%
Carrying power	34 g	42 g	33 g	50 g

This solution was then heated on an electric stove, while being continuously stirred with glass rods. Afterward, the solution was left to cool down to room temperature. Once it had cooled, we added a specific mass (2% w/v) of *B. subtilis* to the solution, ensuring thorough mixing with the microorganisms. We placed the cross-linking solution (6% w/w) in a petri dish, drew the mixture into a 10 mL syringe, and slowly dripped it into the cross-linker solution. This process formed spherical particles with uniform sizes, typically measuring 3–5 mm in diameter. We then cross-linked these spherical particles in the cross-linking solution for 2 h. Following that, we washed the prepared spherical microbe immobilized particles with 0.8% normal saline solution 2–3 times. After storing them for one week at 4 °C, we activated the microbial immobilized particles by soaking them in a 0.8% normal saline solution in a 25 °C incubator for 2 ∼ 4 h.

### Preparation of test solution for particle selection

We used a 600 mg/L and 800 mg/L glucose-glutamic acid (GGA) solution to test and confirm the suitability of the immobilized microbe particles for BOD detection. For each of these solutions, we conducted three sets of parallel experiments for each of the four groups of microbial immobilized particles. Our goal was to determine if these particles could be effectively used in real-world applications by assessing their ability to detect both standard BOD solutions and real water samples. We used the same detection steps for all solutions, employing the COND probe as the detection tool using rapid BOD instrument previously mentioned before.

We used a closed container filled with water samples for the detection process and added microbial immobilized particles to facilitate the reaction. To ensure effective interaction between the immobilized particles and the water samples, we used electromagnetic stirring at a speed of 200 r/min, with stirring intervals of 5 s followed by 30 s of rest. Additionally, a temperature control system was positioned at the bottom of the reactor to maintain a constant temperature of 25 °C throughout the entire reaction. This setup provided optimal conditions for the detection process. The criteria for evaluating the detection ability of microbial immobilized particles are described below.

### Detection accuracy

We calculated the final detection value by averaging six readings taken during a detection period lasting from 3 to 8 min. This final value was then compared with the guaranteed value to calculate the detection error, which assesses the accuracy of the detection method. The detection error is determined by comparing the detected value with the guaranteed value, and the calculation method is as follows:

$$r=\left|\frac{X\;-\;\text{Xa}}{\text{Xa}}\right|\times \;100\%$$

r: detection error,

X: detected BOD value (mg/L),

Xa: guaranteed BOD value of the tested sample (mg/L).

### Detection stability

We assessed the stability of the detection method by calculating the relative standard deviation of the experimental detection values. This value measures the variability or dispersion in the experiments’ detection results and is calculated as follows:

$$S=\sqrt{\frac{\sum_{i=1}^{n}{\left(x_{i}-\bar{x}\right)}^{2}}{n-1}}$$

$$\text{Sr}=\frac{S}{\bar{x}}\times \;100\%$$

S: standard deviation,

Sr: relative standard deviation,

x_i_: BOD value obtained for each test (mg/L),

x: guarantee value of BOD of the test sample (mg/L),

n: number of detections,

i: detection serial number.

## Method validation

### Detection of 600 mg/L GGA standard solution on immobilized particles

**Fig. 2:**
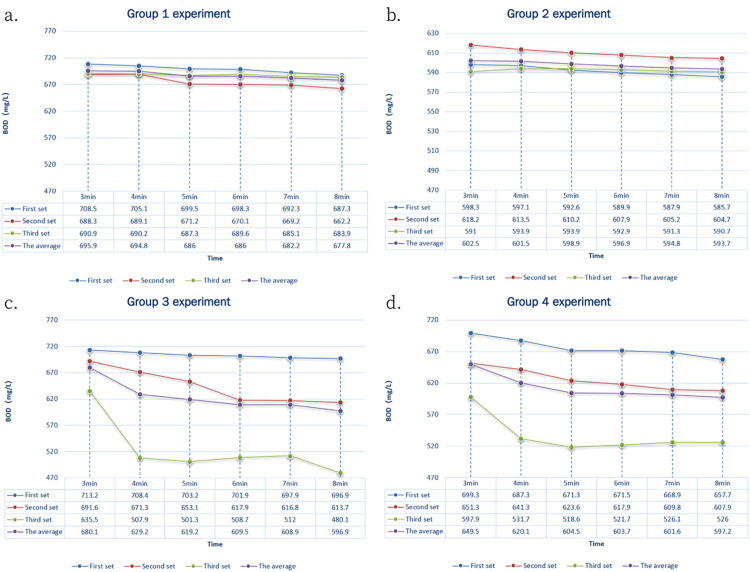
Comparison of test results of 600 mg/L GGA standard solution.

The results showed that the data remained stable for approximately two minutes after the initial test. We then selected the data collected between the 3rd and 8th minutes for comparison to analyse the accuracy and stability of the results. Fig. 2 shows the results of the three sets of parallel experiments for each group of microbial immobilized particles. Each experiment set had six data points, with one data point detected per minute from the 3rd minute to the 8th minute. We calculated the final detection BOD value for each minute as the average of the three sets, and analysed and compared the error and relative standard deviation. The findings are presented in Table 2.

**Table 2:** The test results of 600 mg/L GGA standard solution on immobilized particles.

Analysis	Group 1 (Control)	Group 2	Group 3	Group 4
Final detection BOD value based on average of 6 data points from 3 – 8 mins (mg/L)	687.12	598.05	623.97	612.77
Detection error (%)	14.52	0.32	3.99	2.13
Relative standard deviation (%)	1.81	1.60	13.54	10.21

The results revealed that the control group had a detection error that exceeded the allowable error range (8%) [1], leading to a larger deviation in detection accuracy. The overall order of detection accuracy, based on the detection error, was as follows: Group 2> Group 4> Group 3> Group 1. This suggests that adding various modifiers can enhance the detection accuracy to some extent. When comparing the relative standard deviation, which indicates standard detection stability, the order was as follows: Group 2> Group 1> Group 4> Group 3. This implies that adding a modifier can influence detection stability to some extent. However, it's important to note that different modifiers have varying effects on detection stability. In the concentration range of 500 mg/L to 700 mg/L, adding PVA can improve detection stability to a certain extent. On the other hand, adding diatomite does not significantly enhance detection stability, and adding PAM actually reduces detection stability.

Based on Fig. 2 and Table 2, it was observed that Group 2 experiments exhibited a higher correlation among the trials. In Group 2, each set of parallel experiments showed a more stable trend in detection change. The detected values in Group 2 were notably closer to the guaranteed values of GGA solution (600 mg/L), indicating improved detection reproducibility. Additionally, while Group 4 didn't show a smoother detection trend, its detected value was closer to the guaranteed value of GGA solution compared to Group 1. However, when considering the entire dataset, Group 2 experiments demonstrated better overall stability compared to the Group 1, while Group 3 experiments exhibited lower stability when compared to the dataset as a whole.

### Detection of 800 mg/L GGA standard solution on immobilized particles

**Fig. 3:**
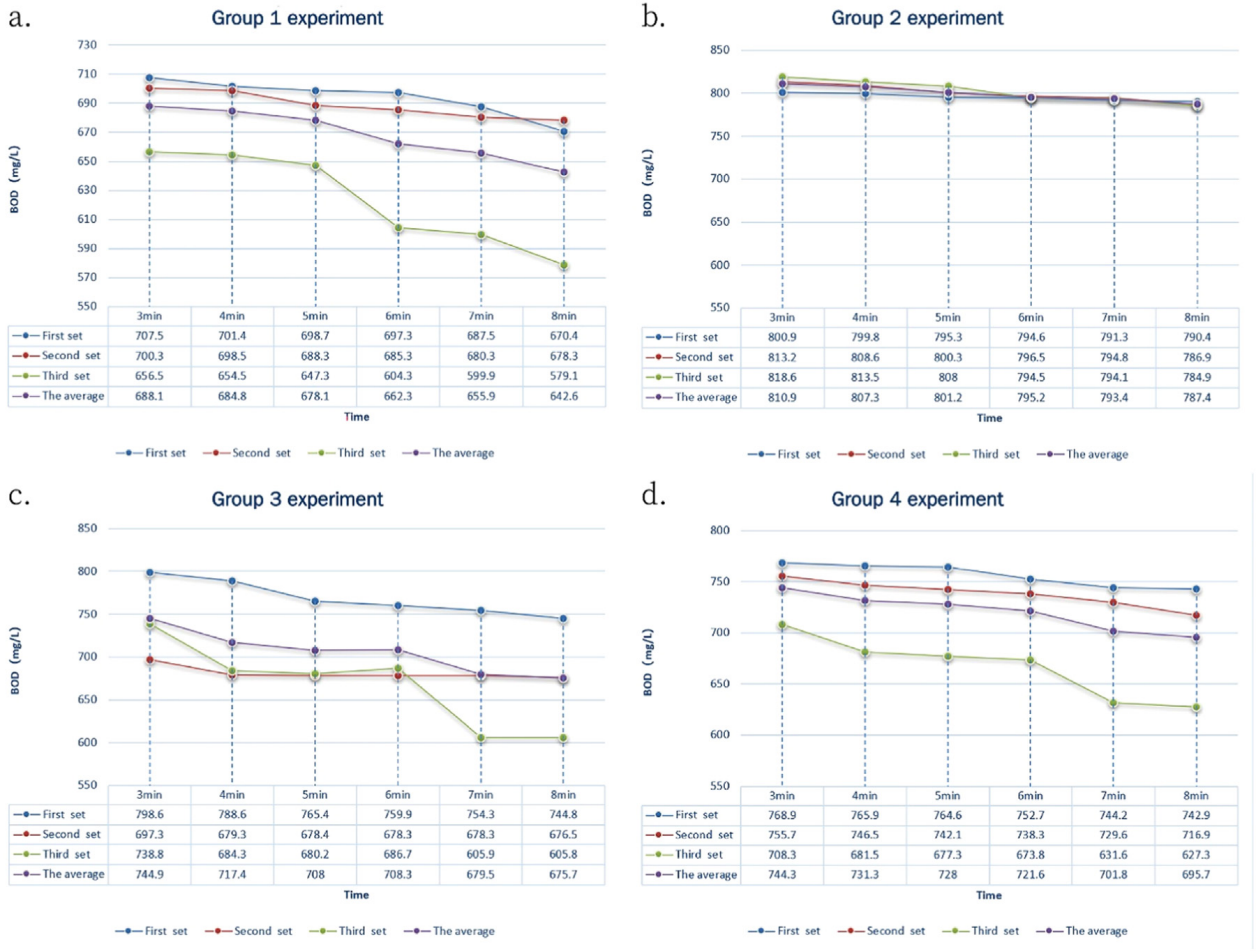
Comparison of the test results of 800 mg/L GGA standard solution.

The results showed that the data remained stable after two minutes of detection. We then selected the data collected between the 3rd and 8th minute for comparison, and analysed the accuracy and stability of the results. Fig. 3 illustrates the results of the three sets of parallel experiments for each group of microbial immobilized particles. Each experiment set included six data points, with one data point detected per minute from the 3rd minute to the 8th minute. We averaged the final detection BOD value for each minute across the three sets, and analysed and compared the error and relative standard deviation, as presented in Table 3.

**Table 3:** The test results of 800 mg/L GGA standard solution.

Analysis	Group 1 (Control)	Group 2	Group 3	Group 4
Final detection BOD value based on average of 6 data points from 3 – 8 mins (mg/L)	668.63	799.23	705.63	720.45
Detection error (%)	16.42	0.10	11.80	9.94
Relative standard deviation (%)	5.75	1.20	7.86	6.17

The results showed that the overall detection accuracy of the 800 mg/L GGA standard solution was similar to that of 600 mg/L GGA standard solution. The order of detection accuracy, based on the overall detection error, was as follows: Group 2> Group 4> Group 3> Group 1. When comparing the relative standard deviation, which reflects detection stability, the order was as follows: Group 2> Group 1> Group 4> Group 3. This suggests that within the concentration range of 700 mg/L to 900 mg/L, adding PVA can improve detection stability. Furthermore, diatomite and PAM exhibited a positive impact on the detection error, albeit their addition led to reduced data stability. The detection experiments revealed that, within the specified concentration range (800 mg/L) all embedding media enhanced stability and reproducibility. (Fig. 3 and Table 3), with particular efficacy was observed in Group 2 and Group 4. Conversely, results from Group 1 indicated a lack of significant improvement in stability and reproducibility compared to these treatments.

## Practical application

### Detection of outsourced BOD standard samples

In this section, we used outsourced BOD standard solution for testing, and all samples were conducted in three tests. We tested five standard samples with varying mass concentrations, each falling within its applicable range. The average test value obtained from the 3 to 8 min detection period served as the result for each test. This result was then compared with the guaranteed value of the sample to assess the accuracy of the test. Sample 1 was collected from a monitoring station in Qingdao, while Sample 2 was purchased from Sichuan China Measurement Standard Technology Co., LTD. Samples 3, 4 and 5 were obtained by diluting Sample 2 by 2 times, 3 times and 20 times, respectively. The test results are shown in Table 4.

**Table 4:** The test results of the outsourced BOD standard samples.

Outsourced BOD solution	Guarantee value (mg/L)	Detection value (mg/L)	Detection error (%)
Sample 1	180	178.9	0.6
Sample 2	210	208.3	0.8
Sample 3	105	104.3	0.7
Sample 4	100	99.2	0.8
Sample 5	10.5	10.2	2.9

The results show that the overall detection error value for the detection of outsourced BOD samples is less than 3%. More specifically, for the highest BOD value (210 mg/L) among these samples, the detection errors was 0.8%, and the lowest (10.5 mg/L) was 2.9%. Overall, for concentrations ranging from 100 to 210 mg/L, the detection error value were less than 1% indicating that the method proposed in this study has good detection capability for standard samples.

### Detection of real wastewater sample

In this part, we compared the effectiveness of the microbial biosensor instrument and the traditional BOD_5_ detection method using the real waste water samples collected from various sources, including domestic sewage from Haibo sewage Treatment Plant, printing and dyeing wastewater from Dong Paper Factory, and gray water from Aosi Agrochemical factory. The test results are shown in Table 5.

**Table 5:** The test results of real wastewater samples.

Types of water samples	Microbial biosensor detection value (mg/L)	BOD_5_ detection value (mg/L)	Detection error (%)
Domestic sewage	98.1	98.9	0.8
Printing and dyeing wastewater	100.7	113.6	11.4
Gray water	71.4	72.3	1.2

The results show that the detection error value for domestic sewage and domestic gray water is less than 2%. However, there is a difference between the detection results obtained through microbial biosensor and the BOD_5_ standard method for the printing and dyeing wastewater. This difference could be due to the presence of more complex chemicals in the printing and dyeing wastewater, which the microbial immobilized particles may not be well adapted to in terms of the water's composition. Wang et al. [2] introduced an innovative reactor-type biosensor for rapid BOD measurement. Their sensor showed a standard deviation of repeatability and reproducibility ranging from ±3.6 to ±6.4%, and ±3.45% to ±4.87%, respectively. Zhang et al. [3] proposed an online BOD measuring instrument based on a microbial membrane reactor. The instrument worked for 4 h, with each water sample BOD test taking about 50 min, and the instrument is in standby state during the rest time. The relative error of the online monitor was ±0.8%, and the relative standard deviation was ±3.2%. In a recent study, Lin et al. [4] proposed an electrochemical biosensor that detected BOD in artificial wastewater and four types of real wastewater with relative errors below 5.4% and 14.6%, respectively. The BOD rapid detection method introduced in this study exhibited better detection accuracy and stability, with results available in just 8 min. This demonstrates that the method meets the requirements for rapid BOD measurement.

## Ethics Statement

This research did not require ethical approval from the committee, as bacteria are not listed among the entities necessitating approval according to the established criteria.

## Declaration of Generative AI and Ai assisted technologies in the writing process

During the preparation of this work, the author(s) used ChatGPT online to rephrase certain words for better clarification. After using this tool/service, the author(s) reviewed and edited the content as needed and take(s) full responsibility for the content of the publication.

## CRediT authorship contribution statement

**Huaihuai Huo:** Conceptualization, Methodology, Data curation, Formal analysis, Writing – original draft. **Jie Li:** Supervision, Writing – review & editing. **Amirah Hurzaid:** Supervision, Data curation, Validation, Writing – review & editing.

## Declaration of competing interest

The authors declare that they have no known competing financial interests or personal relationships that could have appeared to influence the work reported in this paper.

## Data Availability

Data will be made available on request.
